# Supplementary material for: Large-scale discovery of neural enhancers for cis-regulation therapies
Source: bioRxiv. 2025 Nov 5:2025.11.04.686611. Preprint. [Version 1] doi: 10.1101/2025.11.04.686611 (PMC12637570; doi:10.1101/2025.11.04.686611)
Supplement: Supplement 8 [file NIHPP2025.11.04.686611v1-supplement-8.pdf]

bioRxiv preprint doi: https://doi.org/10.1101/2025.11.04.686611; this version posted November 5, 2025. The copyright holder for this preprint (which was not certified by peer review) is the author/funder, who has granted bioRxiv a license to display the preprint in perpetuity. It is made available under aCC-BY 4.0 International license.

Supplementary Figures

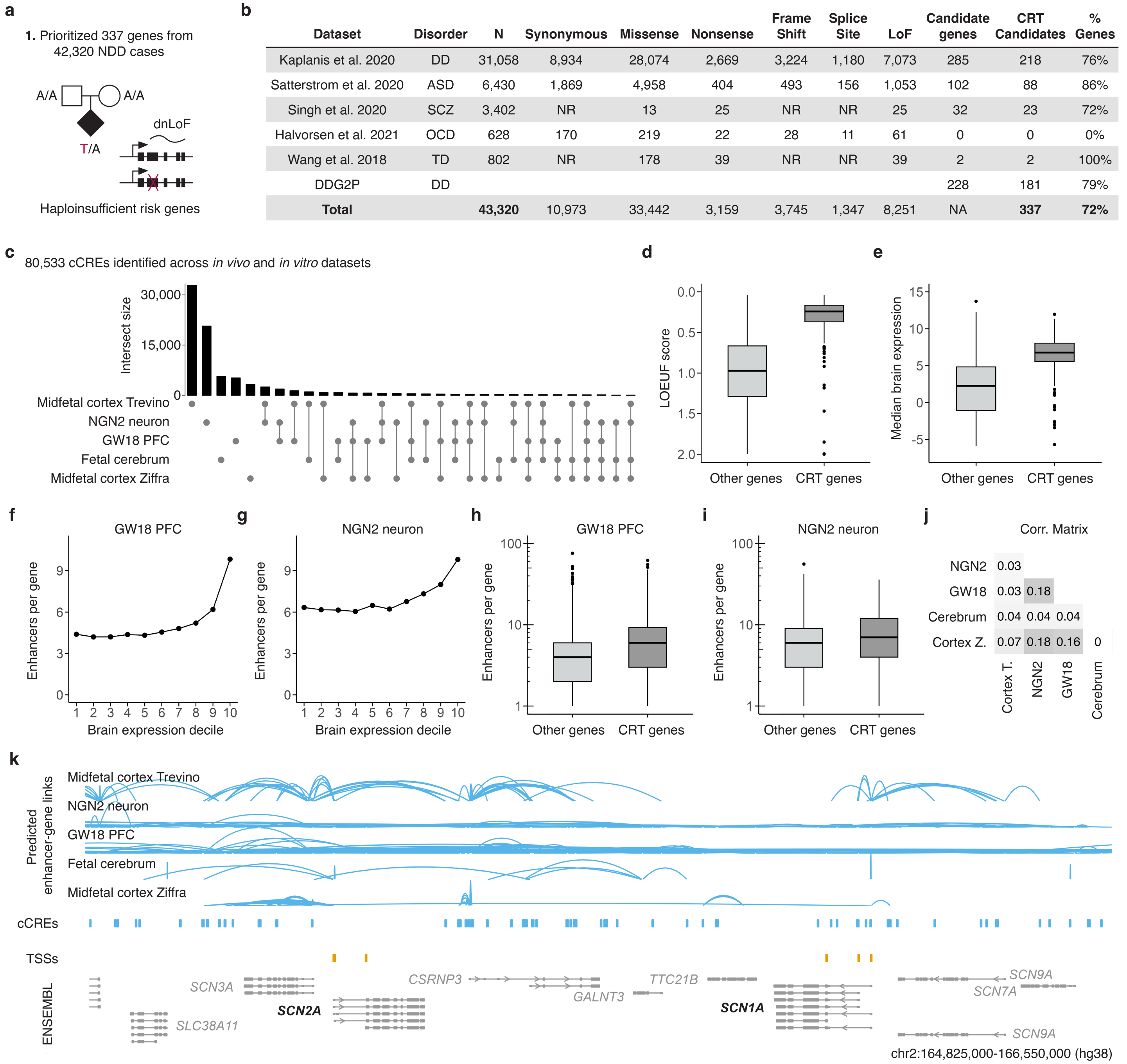

Dataset	Disorder	N	Synonymous	Missense	Nonsense	Frame Shift	Splice Site	LoF	Candidate genes	CRT Candidates	% Genes
Kaplanis et al. 2020	DD	31,058	8,934	28,074	2,669	3,224	1,180	7,073	285	218	76%
Satterstrom et al. 2020	ASD	6,430	1,869	4,958	404	493	156	1,053	102	88	86%
Singh et al. 2020	SCZ	3,402	NR	13	25	NR	NR	25	32	23	72%
Halvorsen et al. 2021	OCD	628	170	219	22	28	11	61	0	0	0%
Wang et al. 2018	TD	802	NR	178	39	NR	NR	39	2	2	100%
DDG2P	DD								228	181	79%
**Total**		**43,320**	10,973	33,442	3,159	3,745	1,347	8,251	NA	**337**	**72%**

Figure S1 | Prioritization of haploinsufficient NDD risk genes and prediction of candidate enhancers. a-b) Prioritization of 337 NDD risk genes from 42,320 cases, sources, and variant counts by class. **c)** Loss-of-function observed/expected upper bound fraction (LOEUF) metrics for 337 prioritized NDD risk genes (labeled CRT) vs. all other genes. **d)** Median brain expression for 337 prioritized NDD risk genes vs. all other genes. **e)** Upset plot illustrates the sources of the 80,533 cCREs identified across *in vitro* and *in vivo* data sets. **f)** Predicted enhancers per gene plotted against deciles of predicted target gene expression in gestational week 18 prefrontal cortex (GW18 PFC). **g)** Predicted enhancers per gene plotted against deciles of predicted target gene expression in iPSC-derived NGN2 neurons. **h)** Predicted enhancers per gene for 337 prioritized NDD risk genes vs. all other genes in GW18 PFC. **i)** Predicted enhancers per gene for 337 prioritized NDD risk genes vs. all other genes in iPSC-derived NGN2 neurons. **j)** Spearman correlation across enhancer prediction approaches. Correlations < 0.005 round to 0 in the visualization. **k)** Tracks for predicted cCREs (blue) and linked TSSs (orange) for all genes in the indicated genomic range are visualized alongside tracks for RefSeq validated transcripts (ENSEMBL/NCBI). Prioritized NDD risk genes are labeled in black.

bioRxiv preprint doi: https://doi.org/10.1101/2025.11.04.686611; this version posted November 5, 2025. The copyright holder for this preprint (which was not certified by peer review) is the author/funder, who has granted bioRxiv a license to display the preprint in perpetuity. It is made available under aCC-BY 4.0 International license.

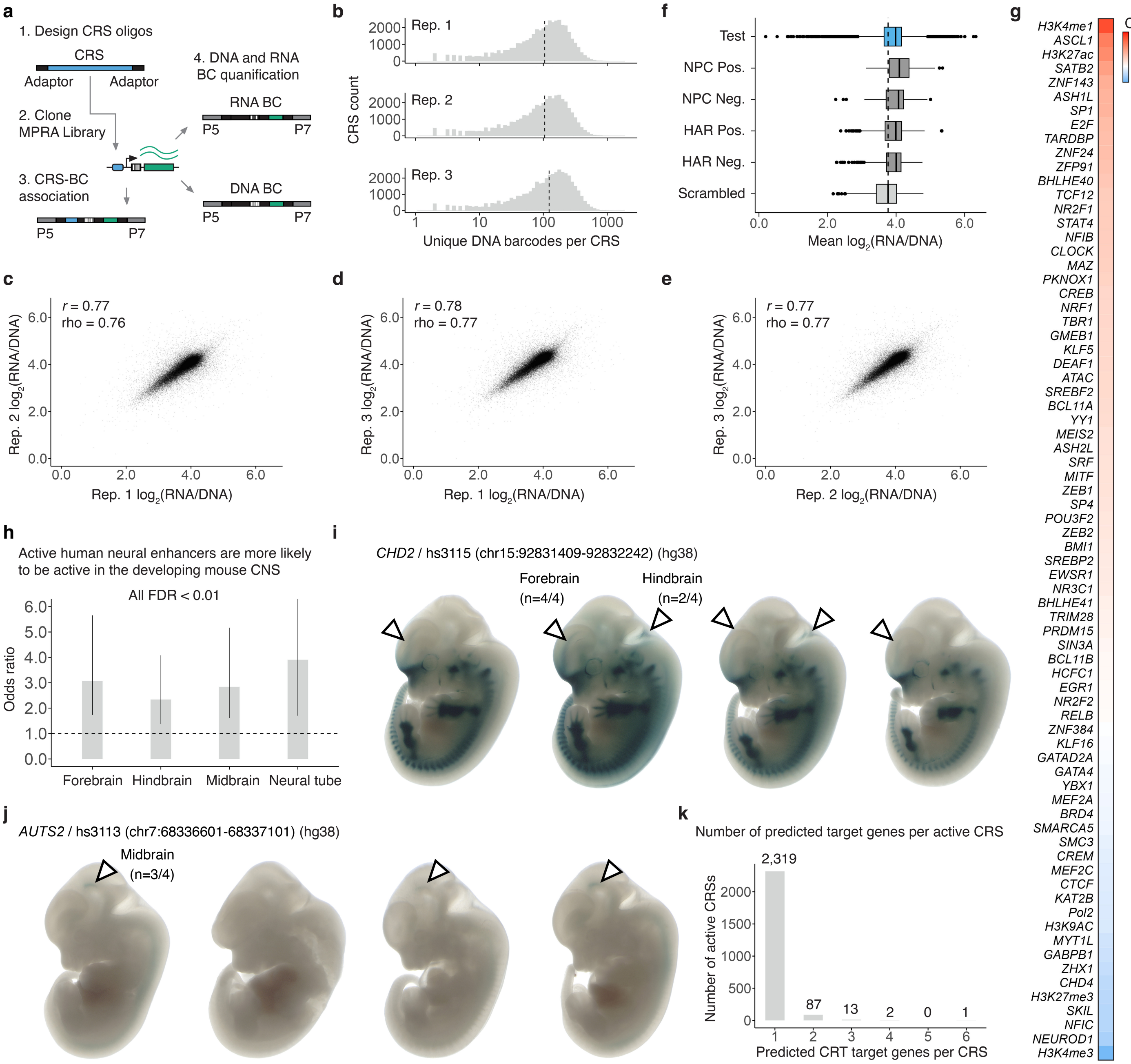

Figure S2 | MPRA design, quality control, and additional results. a) MPRA cloning and sequencing workflow schematic. **b)** Number of unique DNA barcodes recovered per CRS across three transduction replicates. Dashed line represents the median. **c-e)** MPRA activity score correlations across transduction replicates. **f)** MPRA activity score of CRSs grouped by source (**Methods**). Dashed line represents the median of scrambled control sequences. **g)** Correlation between MPRA activity scores and 74 epigenetic/chromatin feature scores derived from neural samples. **h)** Odds ratio of developing mouse CNS reporter expression given MPRA activity. **i-j)** Reporter expression of two MPRA active human enhancers in the developing mouse CNS. Fractions indicate proportion of profiled embryos with detectable expression in the indicated CNS tissue. **k)** Number of predicted target genes per active CRS.

bioRxiv preprint doi: https://doi.org/10.1101/2025.11.04.686611; this version posted November 5, 2025. The copyright holder for this preprint (which was not certified by peer review) is the author/funder, who has granted bioRxiv a license to display the preprint in perpetuity. It is made available under aCC-BY 4.0 International license.

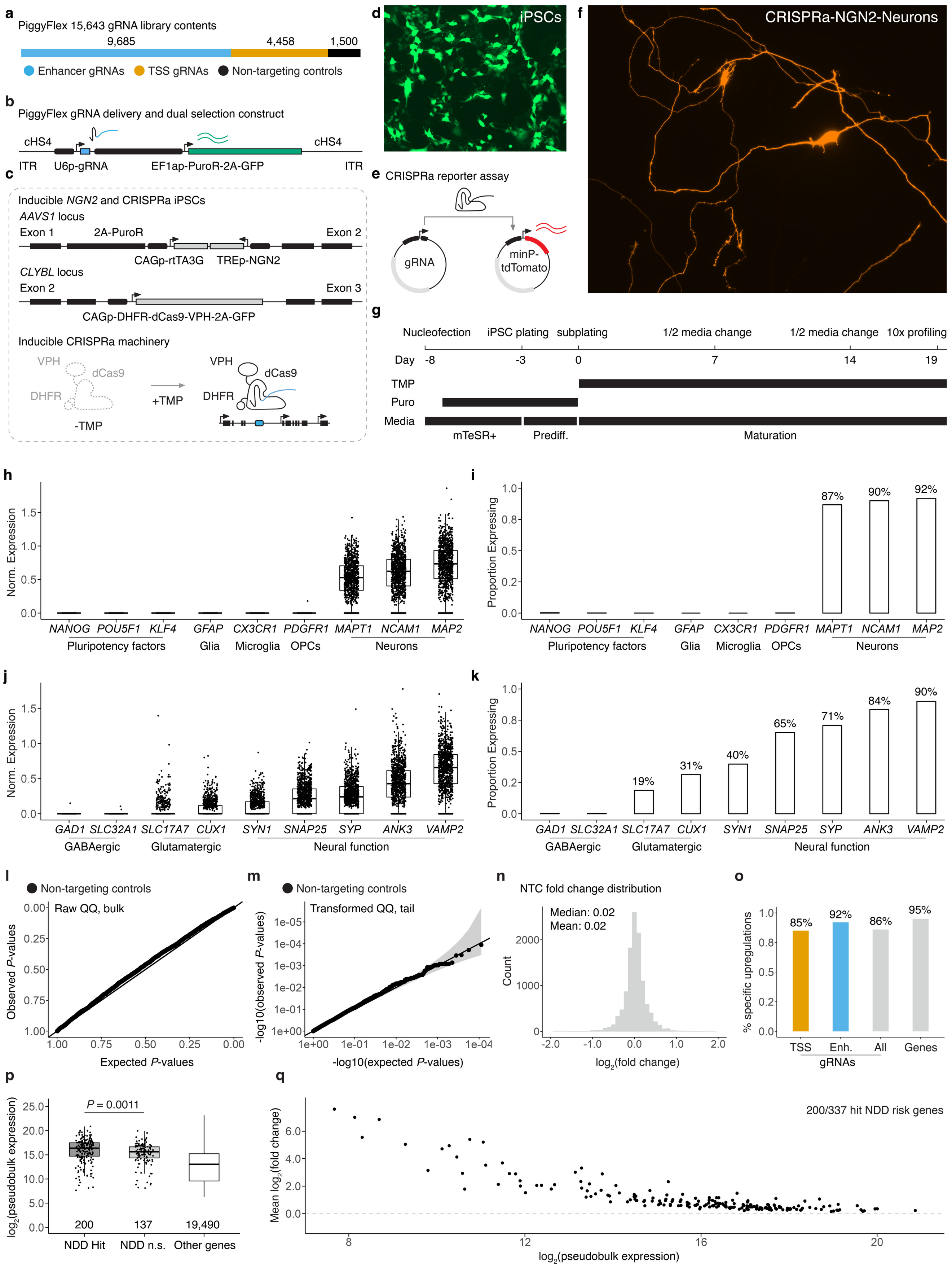

Figure S3 | Multiplex single cell CRIPSRa screen design, quality control, and additional results. a) PiggyFlex gRNA library contents. **b)** PiggyFlex gRNA delivery and dual selection construct. **c)** Integrated, inducible *NGN2* and CRISPRa transgene schematics. Dox-inducible *NGN2* drives neural differentiation from iPSCs. TMP stabilizes/induces functional

bioRxiv preprint doi: https://doi.org/10.1101/2025.11.04.686611; this version posted November 5, 2025. The copyright holder for this preprint (which was not certified by peer review) is the author/funder, who has granted bioRxiv a license to display the preprint in perpetuity. It is made available under aCC-BY 4.0 International license.

CRISPRa machinery. **d)** Selected iPSCs expressing piggyFlex gRNA construct GFP. **e)** minP-tdTomato CRISPRa reporter assay schematic. When co-transfected with the gRNA and reporter plasmids, cells with functional CRISPRa machinery drive strong expression of the otherwise lowly expressed minP-tdTomato reporter. **f)** Differentiated CRISPRa-*NGN2* iPSC-derived neurons transfected with the minP-targeting gRNA and minP-tdTomato reporter drive strong tdTomato expression. **g)** Screen differentiation, selection, and profiling timeline. **h)** Expression of marker genes. Dots represent normalized expression from individual single-cell transcriptomes. Cells are downsampled to 1000 cells for visualization. **i)** Proportion of cells with detectable expression of marker genes. Proportions are calculated on the full set of single cell transcriptomes. **j)** Expression of marker genes. Visualization as in panel **h**. **k)** Proportion of cells with detectable expression of marker genes. Visualization as in panel **i**. **l-m)** Raw and transformed quantile-quantile plots showing distribution of expected vs. observed *P*-values for NTC differential expression tests. **n)** NTC differential expression fold change distribution. **o)** Proportion of upregulations that were target gene specific - i.e. yielded upregulation of only one gene within 1Mb of the target site in *cis*. The first three bars indicate the proportion of TSS-targeting gRNAs (orange), enhancer-targeting gRNAs (blue) and all gRNAs (gray) yielding specific upregulations. The last bar indicates the proportion of the 200 activatable NDD risk genes for which there were one or more specific activating gRNAs identified (190/200, 95%). **p)** Pseudobulk expression levels of NDD risk genes for which an activating gRNA was identified (NDD hit) or not (NDD n.s.). *P*-value is from a Wilcoxon rank-sum test. NDD risk genes are plotted next to all other expressed genes. **q)** Expression level (x-axis) plotted against the average log2 fold-change across activating gRNAs for the 200/337 NDD risk genes.

bioRxiv preprint doi: https://doi.org/10.1101/2025.11.04.686611; this version posted November 5, 2025. The copyright holder for this preprint (which was not certified by peer review) is the author/funder, who has granted bioRxiv a license to display the preprint in perpetuity. It is made available under aCC-BY 4.0 International license.

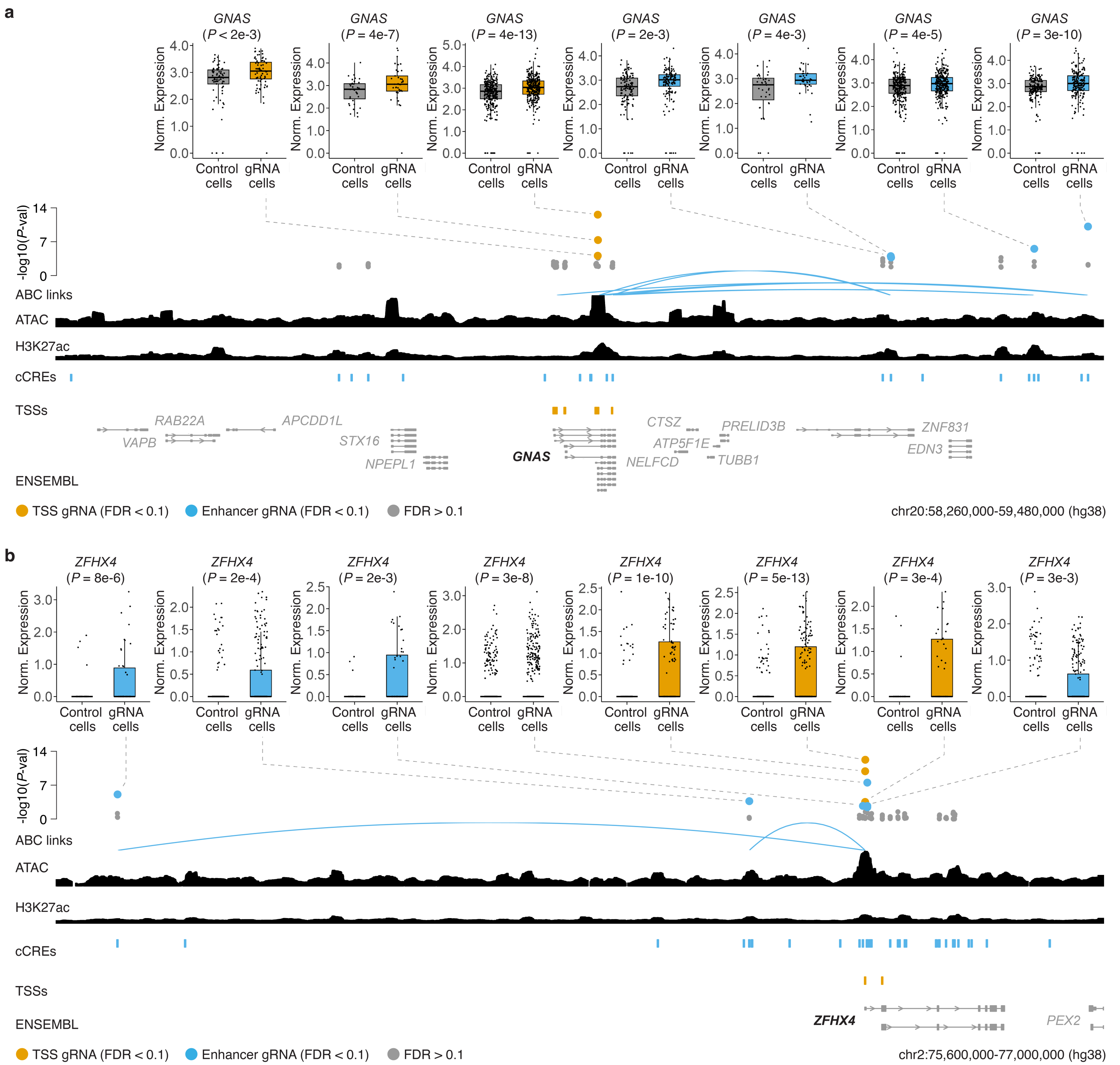

Figure S4 | Highlighted examples of MPRA-active and CRISPRa-responsive enhancers at NDD risk loci. a) (top) Box plots showing log2 fold-change in *GNAS* expression for gRNA cells compared to control cells. Dots represent normalized expression from individual single cell transcriptomes. Control cells are downsampled to have the same number of cells as the indicated targeting gRNA for visualization. (bottom) Multiplex single cell CRISPRa screen differential expression test *P*-values for individual *GNAS* TSS- and enhancer-targeting gRNAs are plotted above tracks for predicted cCREs (blue) and linked TSSs (orange) for prioritized NDD risk genes. TSS- and enhancer-targeting gRNAs yielding significant target gene upregulation (FDR < 0.1) are coloured orange or blue, respectively. These results are plotted above tracks for iPSC-derived neuron ATAC-seq[35], H3K27ac[35], and RefSeq validated transcripts (ENSEMBL/NCBI). Here, only predicted enhancer gene links that were supported by both MPRA and multiplex single cell CRISPRa screens are shown. **b)** *ZFHX4* locus results. Tracks as in panel **a**.

bioRxiv preprint doi: https://doi.org/10.1101/2025.11.04.686611; this version posted November 5, 2025. The copyright holder for this preprint (which was not certified by peer review) is the author/funder, who has granted bioRxiv a license to display the preprint in perpetuity. It is made available under aCC-BY 4.0 International license.

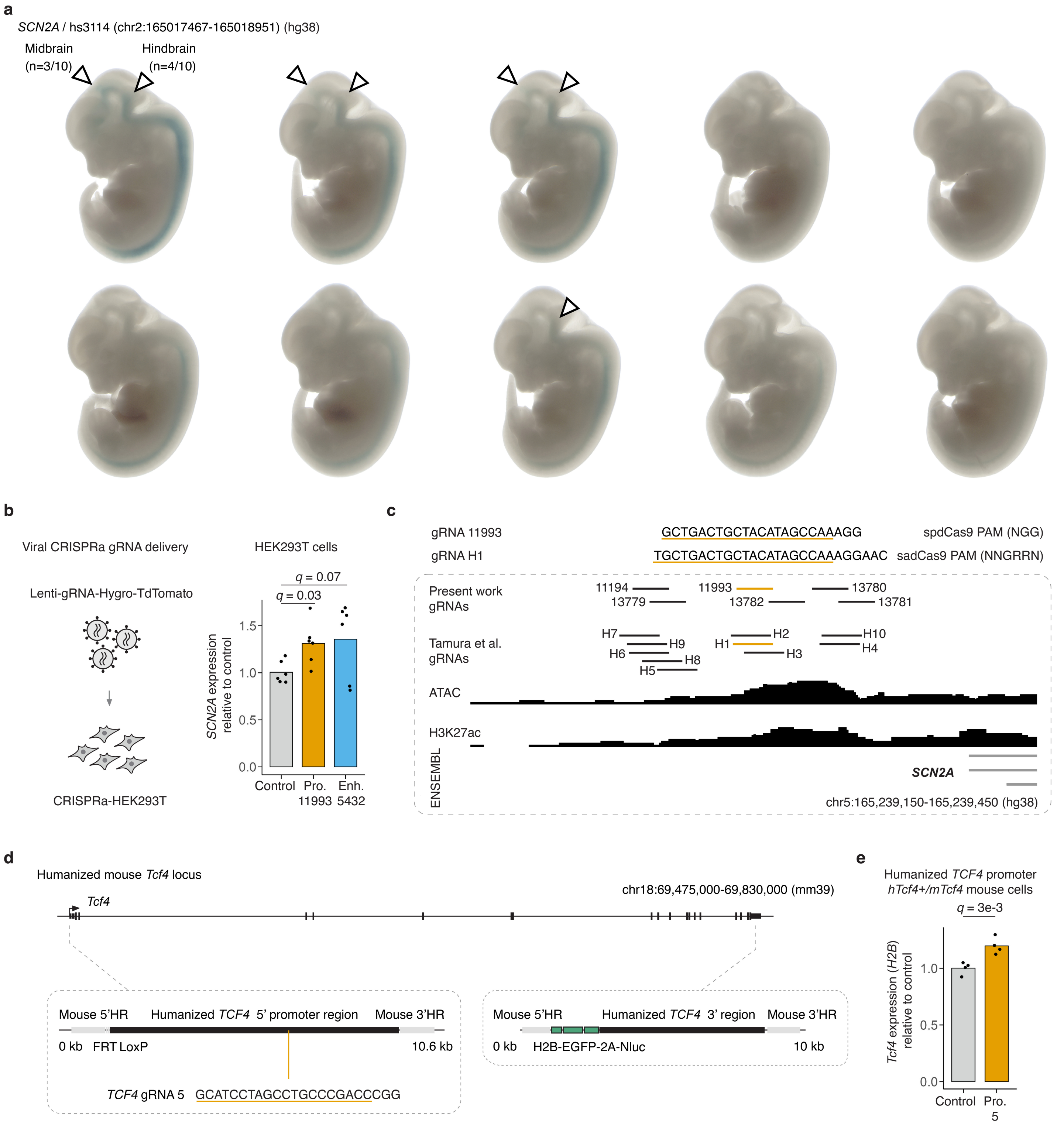

Figure S5 | Additional *SCN2A* and *TCF4* activation and reporter experiments. a) Viral delivery of gRNAs targeting a *SCN2A* TSS and enhancer in HEK293T cells. Bars represent average fold change in *SCN2A* expression (qRT-PCR) relative to control for individual transduction replicates. **b)** Overlap of *SCN2A* TSS-targeting gRNAs tested in the present large-scale screen and a previous one-by-one study using sadCas9. The two strongest activating gRNAs overlap. **c)** Reporter expression driven by a human *SCN2A* enhancer in the developing mouse CNS. Fractions indicate proportion of profiled embryos with detectable expression in the indicated CNS tissue. **d)** Schematic of the engineered mouse *Tcf4* locus with a humanized *Tcf4* 5' and 3' regions. **e)** Viral delivery of a gRNA targeting the *TCF4* TSS in *hTcf4+/mTcf4* mouse fibroblasts with a humanized *TCF4* promoter. Bars represent average fold change in *Tcf4* reporter (*H2B*) expression (qRT-PCR) relative to control for individual transduction replicates.

bioRxiv preprint doi: https://doi.org/10.1101/2025.11.04.686611; this version posted November 5, 2025. The copyright holder for this preprint (which was not certified by peer review) is the author/funder, who has granted bioRxiv a license to display the preprint in perpetuity. It is made available under aCC-BY 4.0 International license.

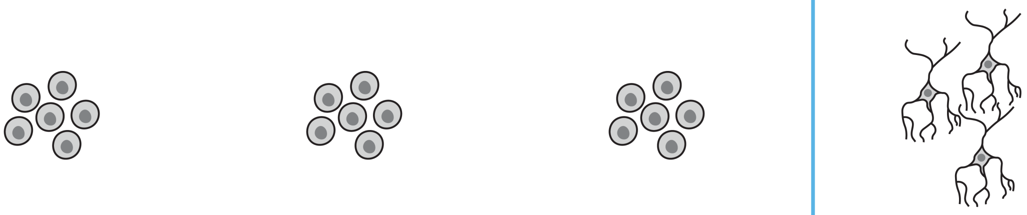

	Nasser et al., 2021	Schraivogel et al., 2020	Gasperini et al., 2019	**Present study**
**E-G Pairs**	88 positive / 3,663	23 positive / 1,283	376 positive / 4,978	91 positive / 2,422
**Profiling method**	FlowFISH	TAP-seq	Perturb-seq	Perturb-seq
**CRISPR modality**	CRISPRi	CRISPRi	CRISPRi	**CRISPRa**
**Cell type**	K562 cells	K562 cells	K562 cells	**iPSC-derived neurons**

Figure S6 | Comparison of large-scale single-cell noncoding CRISPR screens. Comparison of E-G pairs identified out of the number tested for recent large-scale noncoding CRISPR screens. Numbers for the first three CRISPRi datasets collected in K562 cells[31,82,84] are from a recent reanalysis (ENCODE)[83]. Profiling methods, CRISPR modality, and target cell types are also listed.
